# The key factor limiting plant growth in cold and humid alpine areas also plays a dominant role in plant carbon isotope discrimination

**DOI:** 10.3389/fpls.2015.00961

**Published:** 2015-11-03

**Authors:** Meng Xu, Guoan Wang, Xiaoliang Li, Xiaobu Cai, Xiaolin Li, Peter Christie, Junling Zhang

**Affiliations:** ^1^College of Resources and Environmental Sciences, China Agricultural UniversityBeijing, China; ^2^Tibet Agricultural and Animal Husbandry College, Tibet UniversityLinzhi, China

**Keywords:** alpine plants, carbon isotope discrimination, temperature, water availability, key growth-limiting factor

## Abstract

Many environmental factors affect carbon isotope discrimination in plants, yet the predominant factor influencing this process is generally assumed to be the key growth-limiting factor. However, to our knowledge this hypothesis has not been confirmed. We therefore determined the carbon isotope composition (δ^13^C) of plants growing in two cold and humid mountain regions where temperature is considered to be the key growth-limiting factor. Mean annual temperature (MAT) showed a significant impact on variation in carbon isotope discrimination value (Δ) irrespective of study area or plant functional type with either partial correlation or regression analysis, but the correlation between Δ and soil water content (SWC) was usually not significant. In multiple stepwise regression analysis, MAT was either the first or the only variable selected into the prediction model of Δ against MAT and SWC, indicating that the effect of temperature on carbon isotope discrimination was predominant. The results therefore provide evidence that the key growth-limiting factor is also crucial for plant carbon isotope discrimination. Changes in leaf morphology, water viscosity and carboxylation efficiency with temperature may be responsible for the observed positive correlation between Δ and temperature.

## Introduction

Carbon isotope discrimination in plants reflects a range of physiological responses including stomatal conductance, assimilation rate, altered C:N allocation to carboxylation, and leaf structure (Seibt et al., 2008). Water-use efficiency (WUE), which controls the balance between water use and carbon assimilation within plants, is linked to plant carbon isotope discrimination through the substomatal cavities (Farquhar and Richards, 1984). This relationship has thus resulted in numerous studies on plant isotope discrimination in physiological ecology and the global carbon cycle (e.g., Duquesnay et al., 1998; Wang and Feng, 2012; Cernusak et al., 2013).

As is well acknowledged, plant carbon isotope discrimination may be affected by many environmental factors such as temperature, moisture, altitude, latitude, longitude, solar radiation, air pressure, and atmospheric CO_2_ concentration. The fundamental mechanism of how these factors affect plant carbon isotope discrimination is that they can control directly or indirectly the ratio of the intercellular CO_2_ concentration (*c_i_*) to the ambient CO_2_ concentration (*c*_a_). Previous studies have reported that precipitation has a positive and altitude a negative influence on plant carbon isotope discrimination value (Δ), but the effect of temperature varied (e.g., Körner et al., 1988, 1991; Morecroft and Woodward, 1996; Wang et al., 2008, 2013; Diefendorf et al., 2010; Kohn, 2010). Temperature and water availability are considered to be two of the fundamental influential factors. This is based on the observation that variations in altitude, longitude, and latitude can lead to changes in temperature and/or precipitation. Their effects on plant carbon isotope discrimination will therefore be expressed mainly in the effects of temperature and precipitation. Solar radiation and air pressure also vary with altitude, yet their role in the altitudinal trends in plant carbon isotope discrimination are believed to be rather small compared to temperature and/or precipitation (Körner et al., 1988, 1991; Sparks and Ehleringer, 1997; Wang et al., 2008), with the exceptions of Kelly and Woodward (1995) and Zhu et al. (2010) who demonstrated that decreasing Δ with increasing altitude was primarily attributable to decreasing air pressure rather than air temperature. Their conclusion, however, may not always be reliable because the study areas with different elevations that were used to compare air pressure and temperature effects in their study also experience different precipitation inputs. Although the authors claimed no water stress in these study areas, the contribution of precipitation to Δ cannot be ruled out since Diefendorf et al. (2010) found that plant Δ still shows an increasing trend with precipitation when rainfall amount is more than 1000 mm. Marshall and Linder (2013) showed that mineral nutrition may also have a strong effect on plant carbon isotope discrimination. However, Yao et al. (2011) did not observe any change in the Δ of a number of species in response to application of N.

Since carbon isotope discrimination in plants is closely related to plant performance and the key growth-limiting factors play a significant role in plant performance, it has been suggested that the key growth-limiting factor is also the predominant factor affecting plant carbon isotope discrimination (Winter et al., 1982; McCarroll and Loader, 2004). However, as far as we know, this hypothesis has not been confirmed because it is difficult to find a site or region where we know with confidence which environmental factor is the key growth-limiting factor influencing the local plants.

**FIGURE 1:**
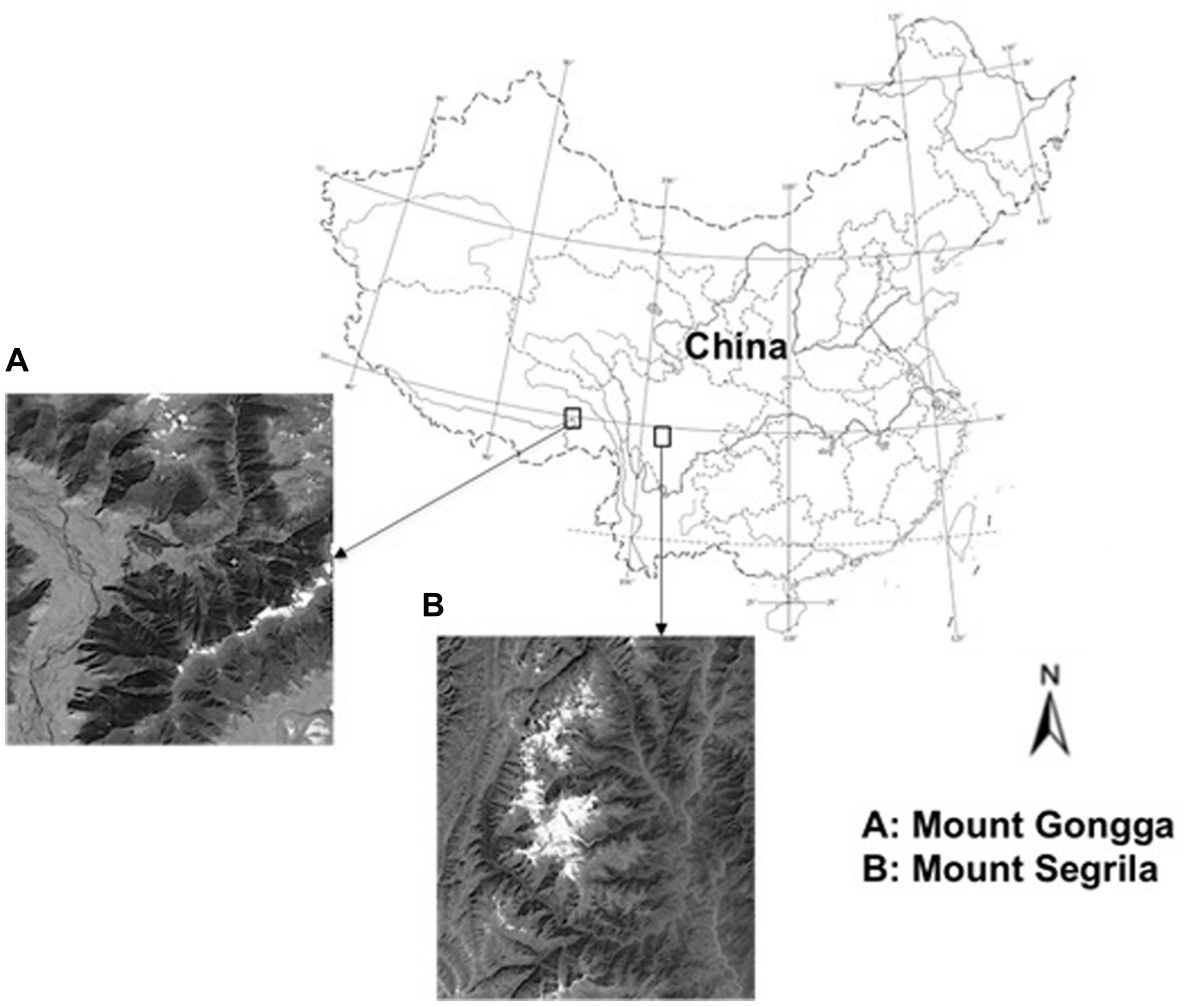
Locations and satellite maps of the studied mountain areas in Qinghai-Tibetan Plateau, China.

In the present study we investigated plant carbon isotope composition (δ^13^C) in two cold and humid montane regions, Mount Gongga and Mount Segrila, both of which are located on the Qinghai-Tibet Plateau (**Figure 1**). As precipitation is abundant in both regions, water availability can be ruled out as a limiting factor, thereby leaving temperature to be the predominant control for growth of local plants. Our objective was to assess whether or not temperature can exert a dominant impact on carbon isotope discrimination of plants growing in cold and humid montane regions.

## Materials and Methods

### Study Area

Mount Gongga is located in the southeast of the Qinghai-Tibet Plateau in Sichuan Province, southwest China (29°20′–30°00′ N, 101°30′–102°10′ E) with considerable differences in terrain and climate between its east and west slopes. The altitude of the east slope ranges from 1100 m (Dadu River valley) to 7600 m above sea level. A continuous vertical vegetation spectrum occurs on this slope, consisting of subtropical evergreen broadleaved vegetation (1100–2200 m, including semi-arid valley with shrubs and grasses below 1500 m, and evergreen broadleaved forests and deciduous broad-leaved forests), temperate coniferous and broad-leaved mixed forests (2200–2800 m), frigid dark coniferous forests (2800–3600 m), alpine subfrigid shrub and meadow vegetation (3600–4200 m), alpine frigid meadow vegetation (4200–4600 m), alpine frigid sparse grass and desert zone (4600–4800 m), and higher altitude alpine ice-and-snow zone (above 4900 m) in sequence. The vertical distribution of soil on the east slope of Mount Gongga is tightly associated with vegetation distribution, and a continuous soil sequence can be observed from 1100 to 4900 m. This consists of yellow-red soil (luvisols; <1500 m), yellow-brown soil (luvisols; 1500–1800 m), brown soil (luvisols; 1800–2200 m), dark-brown soil (luvisols; 2200–2800 m), dark-brown forest soil (luvisols; 2800–3600 m), black mattic soil (cambisols; 3600–4200 m), mattic soil (cambisols; 4200–4600 m), and chilly desert soil (cryosols; >4600 m; Liu and Wang, 2010; Shi et al., 2012). Temperature is certainly the key growth-limiting factor for the plants growing at elevations above 2800 m on Mount Gongga but moisture is definitely not because the climate there is very cold but humid. There are two meteorological observatories (Moxi meteorological observatory, 1640 m above sea level; Hailuogou ecological observatory, 3000 m above sea level) located in the sampling area of Mount Gongga. The mean annual temperature (MAT) and mean summer temperature (MST) above 2800 m are less than 5.2 and 11°C, respectively. However, rainfall is very abundant with a mean annual precipitation (MAP) of 1940 mm at 3000 m, and continues to rise with increasing altitude (Zhong et al., 1997).

Mount Segrila is located on the convergence of the east Nyainqentanglha range and the east Himalaya range, southeast Tibet (29°21′–29°50′ N, 94°28′–94°51′ E). The continuous vertical vegetation and soil spectra of Mount Segrila are described as temperate coniferous and broad-leaved mixed forests (3000–3500 m) with brown soil (luvisols), frigid dark coniferous forests (3500–4200 m) with dark-brown forest soil (luvisols), alpine subfrigid shrub meadow (4200–4500 m) with black mattic soil (cambisols), and alpine frigid meadow and desert zone (4500–5300 m) with mattic soil (cambisols) and desert soil (cryosols). The altitudinal changes in climatic conditions and vegetation spectra were described comprehensively by Xu et al. (2014). There is one meteorological observatories (at 3900 m) located in the sampling area of Mount Segrila; additionally, seven sites with simple meteorological facilities were set in the study area. The MAP and MAT at the elevations above 3100 m are more than 1000 mm and less than 4.2°C, respectively, suggesting that temperature rather than water availability is the predominant growth-limiting factor for local plants (Du et al., 2009).

### Plant Sampling

We collected 457 plant samples in total (444 spermatophytes and 13 pteridophytes) in late August 2004 from the east slope of Mount Gongga (from 1200 to 4500 m). Of these, 181 plant samples (97 plant species in total) were collected from elevations above 2800 m and they are all C_3_ plants. The influence of human activities, sunshine regime, and location within the canopy, was minimized by restricting the sampling to non-shaded sites far from human habitation. Almost all species that we can find at each sampling altitude were collected. At each site 5–7 plants of each species of interest were identified and the same numbers of leaves were collected from each individual. The leaves from each species at each elevation were pooled to give one composite sample. For herbs and shrubs, the uppermost leaves of each individual were taken. For trees, eight leaves were collected from each individual tree and two leaves at each of the four cardinal directions from positions of full-irradiance 8-10 m above the ground surface. Detailed descriptions of the plant sampling on Mount Gongga have been presented previously (Liu and Wang, 2010; Shi et al., 2012).

Leaves were collected at intervals of about 100 m along an elevational transect from 3000 to 4600 m on the west slope of Mount Segrila. The sampling was conducted in late June 2012 when all plants were actively growing at the higher temperatures of the rainy season. Three sampling quadrats, each 50 m × 50 m at each altitude, were set for the plant sampling. Almost all species that we found at each sampling altitude were collected. The uppermost leaves of herbs and shrubs were collected; the leaves of trees were taken from positions 8-10 m above the ground. The leaves from each sampling quadrat at each altitude were pooled to give one composite sample, giving a total of 45 samples from Mount Segrila.

### Soil Sampling and Soil Water Measurement

The samples for soil water content (SWC) measurement were collected in parallel with the plant sampling. The soil sampling on Mount Gongga was conducted in late August 2004 at the end of the rainy season. There was no rain in this study area for at least 1 week based on the meteorological records at the two meteorological observatories. The soil sampling on Mount Segrila was performed in late June 2012 in the rainy season. On Mount Gongga, surface soil samples (0–5, 5–10, and 10–20 cm depth) were obtained for each assigned site (after removing the litter layer) with a soil auger. The soil samples at each locality represented the result of mixing four subsamples randomly taken within a radius of 10 m. On Mount Segrila, three soil cores (2.5 cm diameter, 20 cm depth) were taken randomly from sampling quadrates at each altitude. The soil samples were oven-dried at 105°C to constant weight; the SWC of each sample was the difference between its wet weight and its dry weight divided by its dry weight.

### Carbon Isotope Measurement

All plant samples were oven-dried at 65°C and ground to 60 μm mesh using a steel ball mixer mill MM200 (Retsch GmbH, Haan, Germany). The carbon isotope composition (δ^13^C) of the whole leaf tissue were determined at the Stable Isotope Laboratory of the College of Resources and Environmental Sciences, China Agricultural University, Beijing, China, using a Delta^Plus^XP mass spectrometer (Thermo Scientific, Bremen, Germany) coupled with an elemental analyzer (FlashEA 1112; CE Instruments, Wigan, UK) in continuous flow mode. The elemental analyzer combustion temperature was 1020°C.

The carbon isotopic composition is reported in the delta notation relative to the V-PDB standard. The standard deviation for the δ^13^C measurements is less than 0.152030. Plant Δ is obtained by the following formula based on Farquhar et al. (1989)

$$\Delta =\frac{\delta^{13}C_{air}-\delta^{13}C_{plant}}{1+\delta^{13}C_{plant}/1000}\approx \delta^{13}C_{air}-\delta^{13}C_{plant}$$

in which δ^13^C_air_ is the carbon isotope ratio of atmospheric CO_2_ (-7.8 2030) and δ^13^C_plant_ is the measured δ^13^C value of leaf material.

### Data Selection

Previous studies have reported that photosynthesis and plant enzyme activity can be strongly inhibited when grown at temperatures below 5°C (Graham and Patterson, 1982; Ramalho et al., 2003). Some woody plants cannot withstand much below -6°C for any length of time and cease growth if the maximum daily temperature is below 9°C (Parker, 1963). Based on these studies, we confidently assume that temperature is the key growth-limiting factor for plants grown in cold and humid alpine areas where MAT is below 5°C. Because the MAT below 2800 m is greater than 5.2°C on Mount Gongga (Zhong et al., 1997), the δ^13^C data of the plants grown below 2800 m were excluded in this study. As for Mount Segrila, all δ^13^C data obtained were included in the present study because the MAT values at all sampling sites are less than 4.2°C (Du et al., 2009). Note that the direct measurement of temperature at each elevation is not available, MAT data was obtained by linear interpolation with original data from the meteorological observatories and the simple meteorological facilities in the study areas (Zhong et al., 1997; Du et al., 2009).

### Statistical Analysis

Bivariate correlation analysis was first performed to examine the links between plant Δ and MAT and SWC. Considering the existence of potential interactions between MAT and SWC, partial correlation analysis, in which MAT and SWC were separately controlled, was applied to describe the actual links between plant Δ and MAT and SWC. Regression analysis was used to constrain the influences of MAT and SWC on plant Δ. Since the plant sampling was conducted in two mountain regions and this might introduce a ‘random effect’ (Bolker et al., 2008) into the analysis, a linear mixed model was applied to constrain the influence of MAT and SWC on Δ between the regions, in which MAT, SWC and their interaction (MAT × SWC) are defined as the fixed factor, while the study sites (Mount Gongga or Mount Segrila) are defined as the random factor. Multiple stepwise regression was used to eliminate the influence of potential collinearity existing between MAT and SWC. Variables were selected into the model with *P*-value < 0.05 and excluded with *P*-value > 0.1. The variable with the largest partial correlation coefficient will be first selected into the predicting model. All statistical analysis was performed using IBM SPSS Statistics 22.0 (IBM Corporation, New York, NY, USA).

## Results

### Correlations between Δ and Temperature and Water Availability

The MAT and SWC data and the site-averaged carbon isotope discrimination values (Δ) of plants collected at each sampling site are shown in **Table 1**. In both mountain regions the climate is cold with MAT varying between -5 and 5°C. The surface SWC on Mount Gongga varied from 10.7 to 48.3% with a mean value of 26.7%, while SWC at 0–20 cm depth on Mount Segrila ranged from 25.1 to 76.3% with an average value of 33.1%. There were significant correlations between MAT and SWC on both Mount Gongga (*r* = 0.212, *p* = 0.013) and Mount Segrila (*r* = -0.301, *p* = 0.044).

**Table 1:** Descriptions of climatic condition, dominant vegetation type, and site-averaged plant carbon isotope discrimination value (Δ) of different sampling sites on Mount Gongga and Mount Segrila.

Sampling mountain	Site no.	Altitude (m a.s.l.)	MAT^a^ (°C)	SWC (%)	Site-averaged Δ (2030)	Replicate
Mount Gongga	1	2800	5.2	22.8	21.10	14
	2	2850	4.9	32.5	21.23	4
	3	2860	4.8	32.5	18.26	1
	4	2900	4.6	42.2	21.61	12
	5	3000	4.0	16.7	21.24	12
	6	3100	3.4	34.6	22.36	13
	7	3200	2.8	—	21.21	12
	8	3250	2.5	—	19.71	7
	9	3300	2.2	—	19.31	12
	10	3430	1.4	—	19.45	10
	11	3510	0.94	25.3	20.32	18
	12	3550	0.70	23.7	21.26	7
	13	3600	0.67	22.0	19.62	2
	14	3650	0.10	18.8	18.55	7
	15	3700	-0.20	15.4	20.20	5
	16	3750	-0.50	19.7	18.96	10
	17	3800	-0.80	23.8	17.84	8
	18	3930	-1.58	33.9	18.18	3
	19	3950	-1.7	33.9	18.67	2
	20	4000	-2.0	29.3	17.93	4
	21	4050	-2.3	31.1	18.28	1
	22	4100	-2.6	32.8	18.46	4
	23	4200	-3.2	—	19.04	3
	24	4400	-4.4	30.9	18.75	5
	25	4500	-5.0	18.2	18.74	5
Mount Segrila	1	3135	4.2	33.5	22.25	3
	2	3271	3.3	25.1	21.84	3
	3	3365	2.7	32.1	22.59	3
	4	3456	2.1	45.7	21.93	3
	5	3565	1.4	33.5	22.04	3
	6	3689	0.65	48.7	21.98	3
	7	3770	0.13	76.3	23.06	3
	8	3893	-0.65	56.0	22.37	3
	9	3960	-1.1	36.3	22.59	3
	10	4080	-1.8	71.8	23.23	3
	11	4170	-2.4	45.0	19.94	3
	12	4284	-3.2	50.9	20.86	3
	13	4371	-3.7	48.1	22.18	3
	14	4485	-4.4	36.9	21.61	3
	15	4590	-5.1	46.8	19.49	3


*MAT, mean annual temperature; SWC, soil water content. ^a^MAT data was calculated by linear interpolation with original data from Zhong et al. (1997) for Mount Gongga and Du et al. (2009) for Mount Segrila.*

There was a significantly positive correlation between MAT and Δ of the plants growing on Mount Gongga (*r* = 0.565, *p* < 0.001) and Mount Segrila (*r* = 0.456, *p* = 0.02) in bivariate correlation analysis (**Table 2**), and this impact of MAT on Δ was further expanded after controlling for SWC in partial correlation analysis (*r* = 0.602, *p* < 0.001 for Mount Gongga; *r* = 0.553, *p* < 0.001 for Mount Segrila). The correlation between SWC and Δ, however, was not significant in either mountain region (**Table 2**) and remained non-significant after controlling for MAT, except at Mount Segrila (*r* = 0.400, *p* = 0.007).

**Table 2:** Pearson correlations (*r*) between plant Δ and mean annual temperature (MAT) and soil water content (SWC) on Mount Gongga and Mount Segrila.

	Mount Gongga		Mount Segrila	
		
	*r*	*p*	*r*	*p*
Bivariate correlation				
MAT	**0.565**	**<0.001**	**0.456**	**0.02**
SWC	0.225	0.08	0.202	0.183
Partial correlation				
MAT^a^	**0.602**	**<0.001**	**0.553**	**<0.001**
SWC^b^	0.122	0.158	**0.400**	**0.007**


*Values presented in bold indicated significant correlations (*p* < 0.05). ^a^Indicates controlling for SWC. ^b^Indicates controlling for MAT.*

Considering that the response of carbon isotope discrimation to environmental factors may be dependent on plant functional type (PFT), the influences of MAT and SWC on Δ were analyzed separately based on PFTs. MAT was positively correlated with Δ for all PFTs as well as *Rhododendron* sp., which is a widely distributed evergreen shrub at elevations of 2800–4200 m, as suggested by both bivariate correlation and partial correlation analyses (**Table 3**). By contrast, the correlation between SWC and Δ was not significant in either type of correlation except for shrubs in the bivariate correlation (*r* = 0.289, *p* = 0.044).

**Table 3:** Pearson correlations (*r*) of MAT and SWC with Δ of different plant functional types (PFTs; herbs, shrubs, and trees) as well as *Rhododendron* sp. growing on Mount Gongga.

	Herbs		Shrubs		Trees		*Rhododendron* sp.	
				
	*r*	*p*	*r*	*p*	*r*	*p*	*r*	*p*
Bivariate correlation								
MAT	**0.512**	**<0.001**	**0.575**	**<0.001**	**0.527**	**0.007**	**0.690**	**0.003**
SWC	0.157	0.231	**0.289**	**0.044**	0.251	0.300	0.140	0.682
Partial correlation								
MAT^a^	**0.552**	**<0.001**	**0.603**	**<0.001**	**0.521**	**0.027**	**0.724**	**0.018**
SWC^b^	0.031	0.814	0.170	0.248	0.155	0.540	0.068	0.851


*Values presented in bold indicated significant correlations (*p* < 0.05). ^a^Indicates controlling for SWC. ^b^Indicates controlling for MAT.*

### Regression Analysis and Linear Mixed Model of the Relationship between Δ and MAT and SWC

**FIGURE 2:**
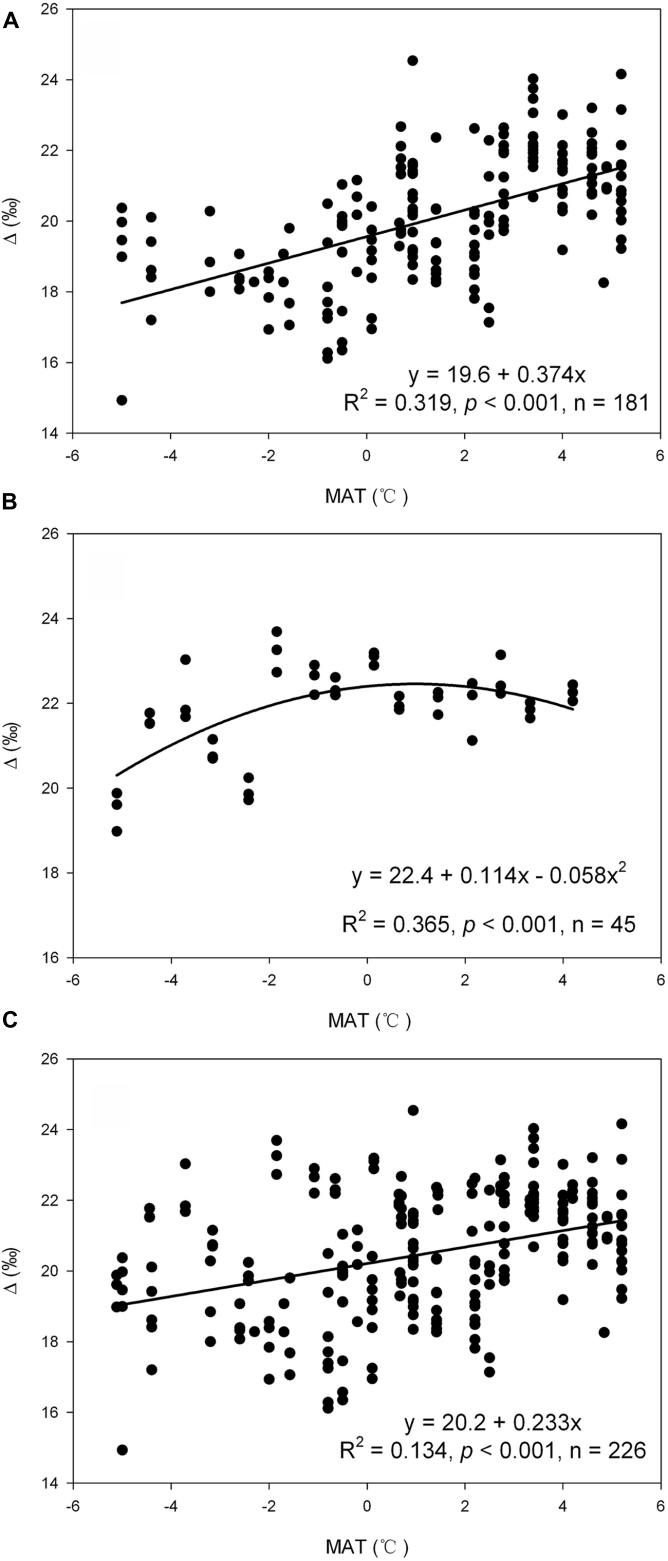
The influence of mean annual temperature (MAT) on Δ as suggested by regression analysis with data in Mount Gongga **(A)**, Mount Segrila **(B)**, and the whole dataset **(C)**.

Results of regression analysis reveal that carbon isotope discrimination was significantly influenced by MAT on Mount Gongga (*R*^2^ = 0.319, *p* < 0.001, **Figure 2A**), whereas the relationship between MAT and Δ is shown as a unimodal pattern with a turning point at MAT = -1°C on Mount Segrila (*R*^2^ = 0.365, *p* < 0.001, **Figure 2B**). Variation in Δ with SWC, however, presents a unimodal pattern on both mountains (Δ = 23.5 - 0.307SWC + 0.006SWC^2^, *R*^2^ = 0.092, *p* = 0.002 for Mount Gongga; Δ = 23.9 - 0.099SWC + 0.001SWC^2^, *R*^2^ = 0.182, *p* = 0.015 for Mount Segrila). When analyzed with the whole dataset, variation in Δ was significantly influenced by either MAT (*R*^2^ = 0.134, *p* < 0.001, **Figure 2C**) or SWC (Δ = 18.9 + 0.053SWC, *R*^2^ = 0.148, *p* < 0.001).

Multiple linear regression analysis shows that MAT and SWC in total accounted for 39.5 and 33.4% of the variance in Δ at Mount Gongga and Mount Segrila, respectively (**Table 4**). Further inclusion of PFTs into the regression model did not increase the estimated R^2^ on Mount Gongga. When calculated with the whole dataset, MAT and SWC altogether accounted for 37.9% of the variance in Δ. In view of the significant correlations between MAT and SWC, multiple stepwise regression analysis was applied to eliminate the influence of collinearity existing between the two variables. The results reveal that MAT was the only variable entered in the stepwise regression model of Δ for Mount Gongga (*R*^2^ = 0.386, *p* < 0.001, **Table 4**), and the first variable selected into the model of Δ for Mount Segrila (*R*^2^ = 0.208, *p* = 0.002) and for the whole dataset (*R*^2^ = 0.169, *p* < 0.001). Both MAT and SWC were finally entered in the model of Δ for Mount Segrila (*R*^2^ = 0.334, *p* < 0.001) and for the whole dataset (*R*^2^ = 0.379, *p* < 0.001).

**Table 4:** Multiple linear regression of plant Δ against MAT, SWC, and PFT.

Model	Variables entered	*R*^2^	Adjusted *R*^2^	*F*	*p*
*Variable selection method: Enter*					
Mount Gongga					
1	MAT+SWC	0.395	0.386	43.721	<0.001
2	MAT+SWC+PFT	0.382	0.362	19.040	<0.001
Mount Segrila					
1	MAT+SWC	0.334	0.303	10.542	<0.001
Whole dataset					
1	MAT+SWC	0.379	0.372	54.635	<0.001
*Variable selection method: Stepwise*					
Mount Gongga					
1	MAT	0.386	0.381	84.789	<0.001
Mount Segrila					
1	MAT	0.208	0.189	11.282	0.002
2	MAT+SWC	0.334	0.303	10.542	<0.001
Whole dataset					
1	MAT	0.169	0.165	36.705	<0.001
2	MAT+SWC	0.379	0.372	54.635	<0.001


In the linear mixed model MAT, SWC and their interaction (MAT × SWC) were defined as the fixed factor and the study sites (Mount Gongga or Mount Segrila) were defined as the random factor. Results show that the sampling mountains did not have any significant effect on the estimated relationship between Δ and MAT or SWC, as the test for the estimated intercept of covariance parameter was not significant (*p* = 0.495, **Table 5**). MAT (*p* < 0.001) and SWC (*p* = 0.002) both significantly affected Δ but their interaction did not (*p* = 0.674).

**Table 5:** Summary of the linear mixed model of Δ with MAT, SWC and their interaction (MAT × SWC) as the fixed variables while the sampling mountains as the random variables.

Linear mixed model results		
**Information criteria**		
Akaike information criterion (AIC)		651.679
Bayesian information criterion (BIC)		658.042
**Estimation of fixed effect**		
*Source*	*F*	*Significance*
MAT	13.475	<0.001
SWC	10.346	0.002
MAT × SWC	0.177	0.674
Intercept	464.366	0.012
**Estimation of covariance parameter**		
*Parameter*	*Wald Z*	*Significance*
Residual	9.407	<0.001
Intercept	0.682	0.495


## Discussion

### Effect of Soil Water Availability on Plant Carbon Isotope Discrimination

The mechanism of water availability on plant carbon isotope discrimination is that the plants would close their stomata to reduce water loss when moisture decreases, resulting in a lower *c_i_*/*c_a_* ratio and thus less negative δ^13^C values. Numerous studies have reported the influence of water availability on plant carbon isotope discrimination (e.g., Wang et al., 2005, 2008; Diefendorf et al., 2010; Kohn, 2010), and a positive correlation between Δ and water availability has been observed on most occasions. In the present study, however, the correlation between Δ and SWC on Mount Gongga was not significant (**Table 2**). It has been observed that carbon isotope discrimination responds differentially over the range of MAP and often becomes nearly constant in wet environments (Kohn, 2010). In the study area of Mount Gongga the water supply is so abundant (with an MAP over 1800 mm) that water availability is thereby no longer a factor limiting plant growth. Our finding is consistent with the results of Diefendorf et al. (2010) that the correlation of Δ with precipitation is not significant when MAP is over 1800 mm.

In contrast to Mount Gongga, no significant correlation was indicated by the bivariate correlation analysis in Mount Segrila. However, we found a significant relationship between SWC and Δ after controlling for MAT in the partial correlation analysis (**Table 2**). The differentiated results from the two sites may derive from the difference in MAP because the MAP on Mount Segrila varies from 980 to 1300 mm, much less than that on Mount Gongga. Nonetheless, this result indicates a partial influence of water availability on carbon isotope discrimination on Mount Segrila. Moreover, the fact that SWC finally entered the model for Mount Segrila as well as for the whole dataset in multiple stepwise regression model of variation in Δ (**Table 4**) also suggests that soil water availability has had an effect on carbon isotope discrimination to some extent. As the present study was conducted in areas with high precipitation where plants obtain sufficient water for their growth, our results suggest that even in humid areas, water availability may still be one of the major determining factors shaping the variation in Δ.

### Temperature as the Key Factor Influencing Plant Carbon Isotope Discrimination

Variation in Δ with changing temperature has been studied extensively (e.g., Körner et al., 1988, 1991; Morecroft and Woodward, 1990, 1996; Hultine and Marshall, 2000; Treydte et al., 2007; Wang et al., 2013). In the present study we also observed a strong impact of MAT on Δ in two mountain regions (**Figure 2**). MAT together with SWC in total accounted for a large proportion of the variation in Δ of the two montane regions (**Table 4**). Although soil water availability is expected to have certain impact on carbon isotope discrimination in the study areas, this impact is more limited than that of temperature as suggested by the results of partial correlation analysis and stepwise regression (**Tables 2** and **4**). Taking these results together, we believe that temperature, rather than soil water availability, has exerted the key influence on carbon isotope discrimination of the plants growing in these two mountain regions. Since temperature is considered to be the key growth-limiting factor in these two frigid alpine areas, the present study supports the hypothesis that the key growth-limiting factor is also the key factor influencing plant carbon isotope discrimination.

Temperature is one of the most important factors that control plant growth and certain physiological processes related to plant gas exchange activity. A decline in temperature usually results in a reduction in enzyme activity and photosynthetic rate (Beerling, 1994), thus leading to decreased CO_2_ assimilation and a lower growth rate as a consequence. Under such circumstances the intercellular CO_2_ concentration (*c_i_*) is likely to increase if the ambient CO_2_ concentration (*c*_a_), stomatal conductance (*g*_s_) and mesophyll conductance (*g*_m_) all hold constant. An increase in Δ with decreasing temperature is therefore expected and has been observed in most of the studies on the influence of temperature on carbon isotope discrimination (e.g., Pearman et al., 1976; McCarroll and Loader, 2004; Treydte et al., 2007; Wang et al., 2013). Our results, however, are inconsistent with these studies and suggest a decline in Δ with decreasing temperature on Mount Gongga (**Figure 2A**). Similarly, several studies have also reported a positive correlation between Δ and temperature along an elevational gradient (Körner et al., 1988; Hultine and Marshall, 2000) and a latitudal gradient (Körner et al., 1991). Moreover, a decrease in Δ with low temperature was also observed in experiments with controlled environment (Morecroft and Woodward, 1990, 1996). There is still no conclusive explanation for this positive correlation but several possible mechanisms have been proposed. One of these is related to the changes in leaf morphology in response to temperature (Körner and Diemer, 1987; Körner et al., 1991). Increased leaf thickness has been observed in alpine plants as an adaptation to low temperatures (Körner et al., 1988; Hultine and Marshall, 2000) and this may cause a longer CO_2_ diffusion distance from the ambient to the intercellular air space and result in a decline in Δ (Körner and Diemer, 1987; Zhu et al., 2010). Kogami et al. (2001) also reported that the plants growing in highlands had lower CO_2_ transfer conductance inside the leaf (*g*_i_) due to greater leaf thickness, thicker mesophyll cell walls and higher mesophyll cell density, resulting in decreased Δ with decreasing temperature. Another explanation is that low temperatures may increase water viscosity (Cernusak et al., 2013). Smith et al. (1984) suggested that lower temperatures might inhibit sapwood water movement and thereby decrease plant water potential, resulting in partial stomatal closure and decreased Δ as a consequence. Finally, the lower Δ with decreasing temperature may also be due to an increase in the efficiency of carbon uptake or carboxylation efficiency at low temperatures, which depends on the amount of active ribulose bisphosphate carboxylase-oxygenase (Rubisco) per unit leaf area (Morecroft and Woodward, 1990).

Changes in temperature and water availability are usually correlated; for example, high temperature can lead to water stress because of high evaporation. These two variables can have synergistic effects on plant growth; co-occurrence of high temperature and water stress was found to constrain plant productivity worldwide (O’Connor et al., 2001). Since carbon isotope discrimination value is an integrated parameter reflecting carbon and water relation, temperature, and water availability are also expected to have interactions on carbon isotope discrimination. Craufurd et al. (1999) found that plant Δ was significantly affected by the interaction of high temperature and water deficit. Xu and Zhou (2005) reported that a perennial grass species decreased its carbon isotope composition in the condition of high nocturnal temperature and water stress. However, the present study showed no significant interaction of temperature and soil water availability on plant Δ (*p* = 0.674, **Table 5**). This result likely suggests that the disturbance of plant water relations by low temperature in the study areas is not significant as reported in previous studies (Norisada et al., 2005; López-Bernal et al., 2015).

## Conclusion

In the present study we conducted an intensive investigation of plant Δ in two cold and humid mountain regions and analyzed the influence of temperature and soil water availability on the variation in Δ. Temperature, the key growth-limiting factor for the local plants, was found to have a significant influence on carbon isotope discrimination irrespective of study area or PFT but the influence of SWC was relatively weak. Future study should consider temporal dynamics on water availability in relation to plant carbon isotope discrimination. Furthermore, a close relationship between plant carbon isotope discrimination and WUE implies that temperature might potentially affect plant WUE. Ecosystems at high altitudes in Tibetan Plateau are fragile and sensitive to climate change. Elevated temperature may reduce WUE in plants, which may have enormous impacts on productivity and stability of ecosystems in the future.

## Conflict of Interest Statement

The authors declare that the research was conducted in the absence of any commercial or financial relationships that could be construed as a potential conflict of interest.
